# Impact of S100A4 Expression on Clinicopathological Characteristics and Prognosis in Pancreatic Cancer: A Meta-Analysis

**DOI:** 10.1155/2016/8137378

**Published:** 2016-01-19

**Authors:** Shanshan Huang, Jiawei Zheng, Yufang Huang, Li Song, Yin Yin, Danzhen Ou, Shangxiang He, Xiong Chen, Xuenong Ouyang

**Affiliations:** ^1^Department of Medical Oncology, Fuzhou General Hospital of Nanjing Military Command, Fuzong Clinical College of Fujian Medical University, Fujian 350025, China; ^2^Department of Medical Oncology, Fuzhou General Hospital of Nanjing Military Command, Medical College Xiamen University, Xiamen 350025, China; ^3^Department of Medical Oncology, Chinese People's Liberation Army 476th Hospital, Fujian 350002, China

## Abstract

*Background.* The small Ca^2+^-binding protein S100A4 is identified as a metastasis-associated or metastasis-inducing protein in various types of cancer. The goal of this meta-analysis was to evaluate the relationship between S100A4 expression and clinicopathological characteristics and prognosis of patients with pancreatic cancer.* Methods*. A comprehensive literature search was carried out in the electronic databases PubMed and Chinese CNKI. Only the studies reporting the correlation between S100A4 expression and clinicopathological characteristics or overall survival (OS) of patients with pancreatic cancer are enrolled. Extracted data was analyzed using the RevMan 5.3 software to calculate the pooled relative risks (95% confidence interval, CI) for statistical analyses.* Results.* Seven studies including a total of 474 patients were enrolled into this meta-analysis. Negative expression of S100A4 was significantly associated with higher 3-year OS rate (RR = 3.92, 95% CI = 2.24–6.87, *P* < 0.0001), compared to S100A4-positive cases. Moreover, negative expression of S100A4 was also related to N0 stage for lymph node metastasis (RR = 2.15, 95% CI = 1.60–2.88, *P* < 0.0001). However, S100A4 expression was not significantly correlated with histological types and distant metastasis status.* Conclusion.* S100A4 expression represents a potential marker for lymph node metastasis of pancreatic cancer and a potential unfavorable factor for prognosis of patients with this disease.

## 1. Introduction

Pancreatic cancer (PC) is one of the most aggressive and highly lethal types of cancer worldwide. The 5-year survival rate of patients with PC is less than 5%, and only 15–20% PC patients are eligible for curative surgery when they are first diagnosed [1, 2]. This poor outcome is mainly due to its high propensity for locoregional invasion and early development of distant metastases [3]. In addition, lack of biomarker for tumor metastasis as a prognostic indicator of PC patients also represents a major contributor to the high mortality of this malignancy. While the tumor marker carbohydrate antigen 19-9 (CA19-9) and carcinoembryonic antigen (CEA) often correlate with therapeutic response and tumor recurrence, they have neither sensitivity nor specificity [4]. Therefore, the identification of valid, reliable biomarkers for the prediction of the prognosis of PC patients is of great importance for clinical management of this disease.

S100 protein, as it is named, was first identified in the S100 soluble fractions purified from bovine brain [5]. Later, S100 proteins were characterized as a family of multiple calcium-binding proteins that contain two EF-hand Ca^2+^-binding motifs, which are involved in a variety of physiological functions via calcium-dependent interaction with numerous target proteins [6]. Of note, whereas S100 proteins are commonly upregulated in many cancers, the S100 family also plays important roles in tumor progression of various types of cancer [7]. S100A4 (also named as calvasculin, mts-1, pEL-98, 18A2, p9Ka, CAPL, Fspl, etc.), a member of the S100 protein family, was defined to be engaged in tumor invasion and metastasis [8], but not oncogenesis* per se* as S100A4 transgenic mice do not develop tumor. Such a distinct feature of S100A4 in promoting tumor metastasis, thereby also known as metastasin [7], makes it a strong candidate as a biomarker for predicting disease progression, particularly tumor metastasis, and clinical prognosis [9]. Indeed, S100A4 is highly expressed in many types of cancer such as breast cancer [10, 11] and particularly gastrointestinal cancers, including colorectal [12–14], gastric [15, 16], esophageal [17, 18], and pancreatic cancer [19]. Moreover, S100A4 expression has correlated with tumor metastasis and poor prognosis of patients with several types of cancer [15, 20, 21], including PC [22].

However, unlike colorectal [12], gastric [23, 24], lung cancer [25], no meta-analysis, to the best of our knowledge, has been carried out so far to analyze the relationship between S100A4 and clinical outcomes of patients with PC. Here, we report a meta-analysis of the current literatures to address correlation between S100A4 expression and clinicopothological features or patient survival in PC.

## 2. Materials and Methods

### 2.1. Search Strategy

A comprehensive literature search was performed using the electronic databases PubMed and Chinese CNKI. The search strategy used for PubMed was “(pancreatic cancer [Title/Abstract]) OR (pancreatic carcinoma [Title/Abstract]) OR (pancreatic neoplasms [Title/Abstract]) OR ((“Neoplasms”[Mesh]) AND (“Pancreatic Neoplasms” [Mesh]))” AND “(S100A4 [Title/Abstract]) OR (S100 [Title])”. There was no limitation on race/ethnicity, gender, or language or year of publication. A similar search strategy in Chinese terms was used for CNKI.

### 2.2. Selection Criteria

The studies were eligible only if they investigated S100A4 expression in primary PC tissue (surgical or biopsy) of patients and if they met at least one of the following two criteria: (a) used overall survival (OS) as an endpoint and (b) used clinicopathological characteristics as investigative parameters. The studies were excluded if their data was not sufficient to determine an estimate of pooled relative risk (RR) and 95% confidence interval (CI). If there were multiple or overlapped publications on the same patient population, only the one(s) reported in English or most recently were included.

### 2.3. Data Extraction

Two authors (Shanshan Huang and Jiawei Zheng) independently performed filtering and quality assessment of the selected literatures. Disagreement was resolved through independently extracting data from the original article by the third author (Yufang Huang), and consensus was reached by discussion. The following data were extracted from each selected study: first author's last name, year of publication, country of the population studied, number of cases, cutoff value for the definition of S100A4-positive expression, duration of follow-up, N category of lymph node metastasis, M category for distant metastasis, tumor histology, and S100A4 expression-related OS rate. For the articles in which prognosis was plotted only as the Kaplan-Meier curves, the Engauge Digitizer V4.1 (http://getdata-graph-digitizer.com/) was then used to extract survival data.

### 2.4. Statistical Analysis

The RevMan 5.3 software (Cochrane Collaboration) was employed to perform the meta-analysis. Comparison of dichotomous measures was made to estimate the pooled relative risk (RR) and its 95% confidence interval (CI). Statistical heterogeneity was assessed using the Chi-square test and *I*-square test. According to the absence of presence of heterogeneity, random effects model or fixed effects model was used to merge the RR, respectively. Sensitivity analysis was conducted to determine if certain single article could influence the overall result. Due to the small number (<10) of the studies eligible for the meta-analysis, publication bias was not assessed.

## 3. Results

### 3.1. Study Selection

**Figure 1:**
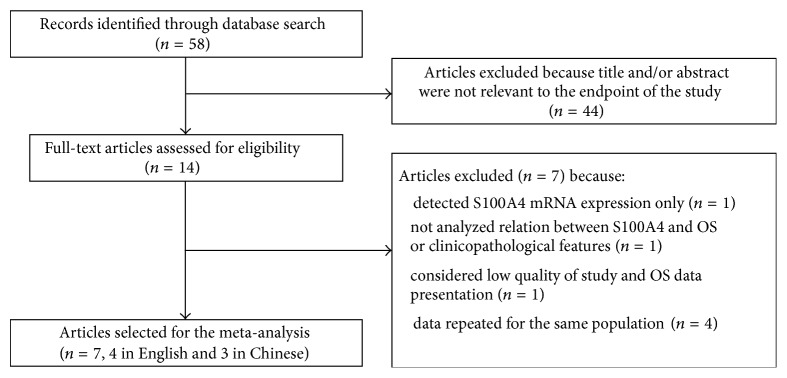
Flow chart for selection of the included studies.

As shown in Figure 1, 58 records were initially retrieved using the predefined search strategy. After browsing the retrieved titles and abstracts, 44 records were excluded due to no relevant endpoint provided. The remaining 14 records were downloaded as full-text and carefully accessed one by one. Among them, 6 studies were excluded, including one that only examined S100A4 expression at mRNA level [32], one that did not analyze the relation between S100A4 expression and OS or clinicopathological features [33], and four duplicated reports on the same study population [29, 34–36]. As a result, 8 published studies met the inclusion criteria. However, when performing data extraction, one eligible study [37] was further excluded due to low quality of the study (e.g., inconsistency of data) and publication that caused failure of extracting survival rate from the Kaplan-Meier curve presented. Therefore, 7 studies including 474 patients who were all diagnosed as pancreatic ductal adenocarcinoma (PDAC) were finally selected for the meta-analysis.

### 3.2. Study Characteristics

Characteristics of 7 selected studies [19, 26–28, 30, 31, 38] were summarized in Table 1. Among them, 4 studies were published in English while 3 in Chinese. Of note, all studies were conducted in Asian patient population, including 1 in Korea, 2 in Japan, and 4 in China. For the methods used to detect S100A4 expression, 5 studies performed immunohistochemical analysis on whole tissue sections, and the other 2 used tissue microarray [19, 28]. S100A4 positivity was defined by both distribution of positively stained cells and intensity of staining in 5 studies, but one only by distribution of positively stained cells [19], while one did not provide the definition for S100A4 positivity [26].

### 3.3. S100A4 Expression and 3-Year OS of Patients with PDAC

**Figure 2:**
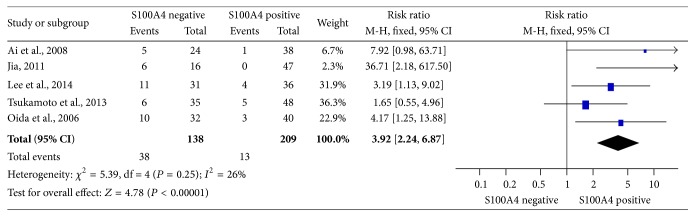
Relation between S100A4 expression and 3-year OS rate. Comparison was made between S100A4-negative and S100A4-positive expression for 3-year OS rate.

Overall, 3-year OS rate was reported either directly or by the Kaplan Meier curves in 5 studies including 347 patients. Notably, the meta-analysis revealed that, S100A4-negative expression was significantly associated with better 3-year OS rate, compared to its positive counterpart (Figure 2, RR = 3.92, 95% CI = 2.24–6.87, *P* < 0.00001, fixed effects model), with 3.92-fold higher 3-year OS rate of patients with S100A4-negative* versus* S100A4-positive PDAC tumors.

### 3.4. S100A4 Expression and Clinicopathological Features of PDAC

**Figure 3:**
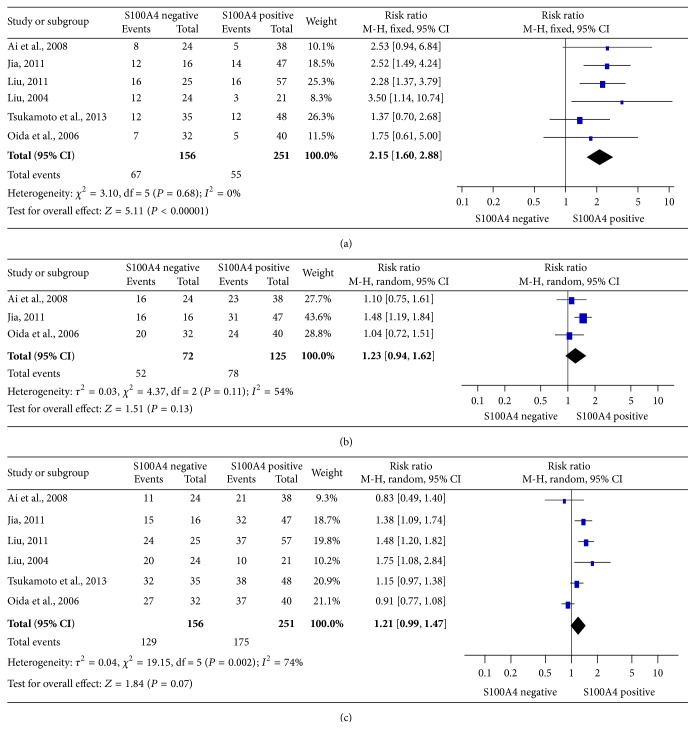
Relation between S100A4 expression and clinicopathological features of PDAC. (a) Relation between S100A4 expression and lymph node metastasis (N category). Comparison was made between S100A4-negative and S100A4-positive expression for N0 status. (b) Relation between S100A4 expression and distant metastasis (M category). Comparison was made between S100A4-negative and S100A4-positive expression for M0 status. (c) Relation between S100A4 expression and tumor histology. Comparison was made between S100A4-negative and S100A4-positive expression for well or moderately differentiated tumor cells.

6 studies reported data on the relation between S100A4 expression and N category of PDAC tumor. There was a significant association between S100A4-negative expression and N0 lymph node metastasis (Figure 3(a), RR = 2.15, 95% CI = 1.60–2.88, *P* < 0.00001, fixed effects model). However, S100A4 expression was not related to distant metastasis (Figure 3(b), RR = 1.23, 95% CI = 0.94–1.62, *P* = 0.13, random effects model) in 3 eligible studies or tumor histology (Figure 3(c), RR = 1.21, 95% CI = 0.99–1.47, *P* = 0.07, random effects model) in 7 eligible studies.

### 3.5. Sensitivity Analysis and Publication Bias

**Figure 4:**
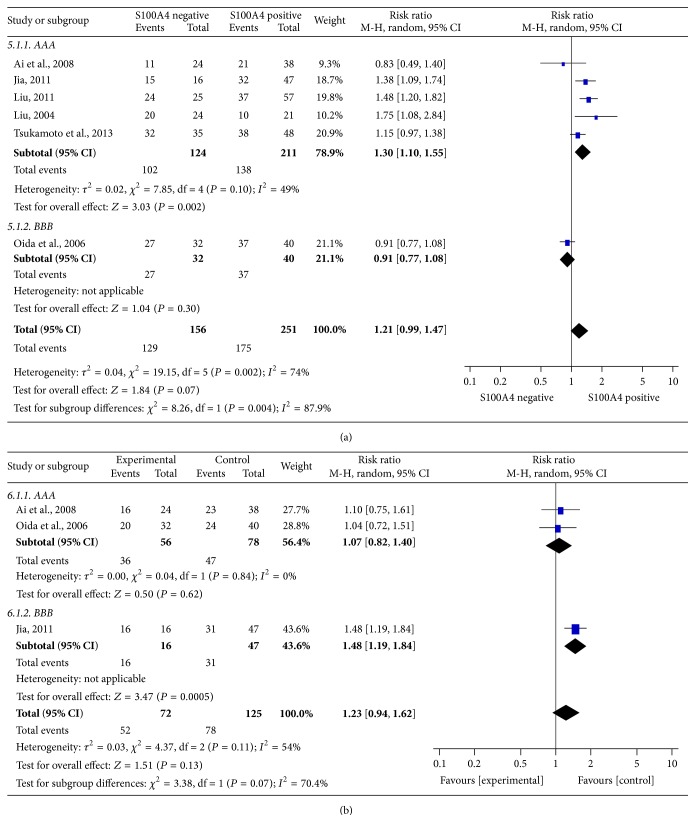
Sensitive analysis. (a) Sensitive analysis for tumor histology group. (b) Sensitive analysis for distant metastasis group.

Sensitivity analyses were further performed to determine the robustness of the results described above. For the statistically significant correlations between S100A4 expression and the 3-year OS rate or N0 lymph metastasis, the results were not altered by deletion of any single study (data not shown). However, final *I*-square score or pooled RR score about the relations between S100A4 expression and tumor histology or distant metastasis was largely affected by deletion of the study by Oida et al. [26] (Figure 4(a)) or Jia et al. [38] (Figure 4(b)). The total number of the studies (<10) included in this meta-analysis was too small to access publication bias as the default publication bias already existed.

## 4. Discussion

**Figure 5:**
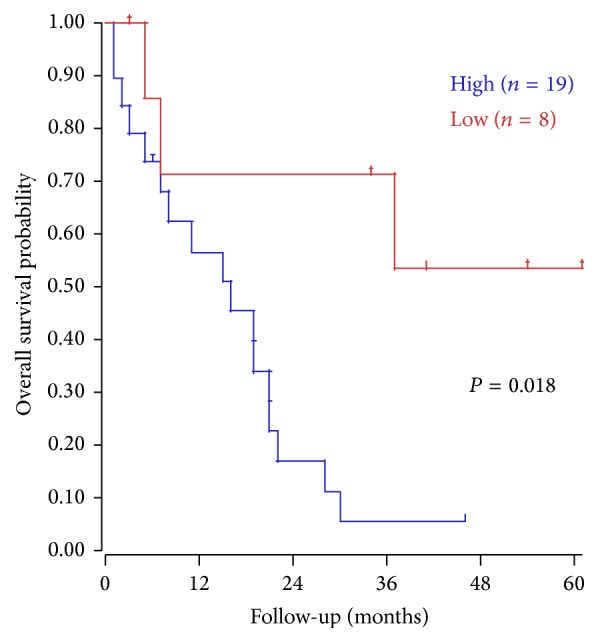
Correlation between S100A4 expression and overall survival. Kaplan-Meier analysis (Mixed pancreatic PDA – Sadanandam – 47) was performed using R2: Genomics Analysis and Visualization Platform (http://r2.amc.nl/).

Due to its aggressiveness and poor prognosis, PC poses a heavy burden especially in North America [3]. Currently, CA19-9 and CEA are the most widely used markers in gastrointestinal malignancies. However, due to its low sensitivity and specificity, their secretion rates from individual tumors and nonspecific elevations impair their tumor marker utility and call for the development of additional reliable marker for PC. In this meta-analysis, S100A4 was considered to be a promising candidate. In our study, a combined analysis of 7 clinical researches, which detected the S100A4 antigen in PC tissues, revealed a dismal prognostic outcome in patients with S100A4-positive staining. To further validate our results, a web based analysis was performed using R2: Genomics Analysis and Visualization Platform (http://r2.amc.nl/), which revealed a significant correlation between S100A4 gene expression and overall survival of patients with PC (Figure 5, *P* < 0.02 for high versus low expression, expression cutoff: 152.1 [minimal group = 8]).

What makes S100A4 contribute to the poor prognosis in PC? On the one hand, S100A4 is commonly found upregulated in various kinds of cancer cells, including PC cells [9]. On the other hand, S100A4 mechanistically acts to bind to multiple proteins, including (a) cytoskeletal proteins (e.g., actin, tubulin, and tropomyosin) to directly regulate cytoskeletal rearrangement and cell motility, probably involved in cancer cell invasion; (b) MDM2 to promote degradation of the tumor suppressor p53; (c) EGFR ligands to enhance EGFR/ErbB2 receptor signaling and cell proliferation; (d) heparan sulfate to activate a Galphaq-coupled receptor, thereby regulating cell apoptosis and differentiation; (e) receptor for advanced glycation end-product (RAGE) to induce cancer cell motility, likely via activation of MAPK/ERK and hypoxia signaling [39–41]. In the present study, we also found that S100A4 expression is significantly correlated with lymph node metastasis. Previous studies have shown that S100A4 expression can upregulate the matrix metalloproteinases (MMPs), which play critical role in tumor metastasis through degradation of extracellular matrix (ECM), including MMP-9, MMP-13, and MMP-2 [22]. In addition, S100A4 protein can also lower the expression of E-cadherin, an inhibitor of MMPs, to promote cell invasion and metastasis [42]. Conversely, blockade of S100A4 (e.g., by a function-blocking anti-S100A4 monoclonal antibody) prevents metastasis burden* in vivo* [43, 44]. All these studies reflected the strong association of S100A4 with PC metastasis. Pooled data also suggested a trend towards positive expression of S100A4 which was associated with low degree of tumor differentiation and status of distant metastasis, though statistically significantly it was not reached. The small sample size and the missing of some relevant information might be the reasons that no association of S100A4 positivity with histology or metastasis was observed. Generally speaking, S100A4 could be a marker for poor prognosis and lymph node metastasis of PC.

Given the strong correlations between S100A4 expression and prognosis/clinicopathological features, it might be helpful in the development of approaches to PC. And* in vitro* test has shown that S100A4 activates Src-FAK-mediated dual signaling pathways, promoting PC progression [45]. Further, enforced expression of S100A4 increased cell movement [46] and invasion [47], whereas siRNA S100A4 knockdown suppresses cell mobility [48]. Hence, S100A4 could be a potential target for PC therapy. In addition, Mahon et al. [49] have demonstrated that the knockdown of S100A4 expression can lead to an increased sensitivity of PC cell lines to gemcitabine treatment. We infer that in the case of PC, S100A4 inhibitor could improve survival and prognosis. Nevertheless the clinically translational potentials require deeper investigation.

Our results should be interpreted cautiously since several limitations exist in the present study. Firstly, the number of the eligible published studies, as well as the number of patients enrolled in each of these studies, is relatively small. Secondly, all the included studies involve only the Asian population, which most likely cannot reflect whether S100A4 expression would correlate with prognosis of PC in the European population. Thirdly, the data for baseline measurement of clinicopathological characteristics are not accessible in some included studies. Moreover, the cutoff values for defining S100A4 expression vary between the included studies. Therefore, further high-quality studies with large sample size are needed to draw a definitive conclusion on S100A4 as a biomarker for progression of PC. More importantly, an improved knowledge in S100A4 expression and cancer biology can further potentiate the emergence of new targeted therapies.

## Figures and Tables

**Table 1:** Characteristics of the included studies (P: S100A4-positive; N: S100A4-negative; NA: not available).

Author	Year	Country	Number of patients	Duration of follow-up	Cutoff scores (positive/negative)	N category (N0/N1)	Distant metastasis (M0/M1)	Histology (well, mod/Por)	3-year OS rate
Oida et al. [26]	2006	Japan	72	NA	>NA (40/32)	P (5/35) N (7/25)	P (24/16) N (20/12)	P (37/3) N (27/5)	P 7.5% (3/40) N 31.3% (10/32)
Ai et al. [27]	2008	China	62	NA	Score *⩾* 2 (38/24)	P (5/33) N (8/16)	P (23/15) N (16/8)	P (21/17) N (11/13)	P 2.1% (1/38) N 20.8% (5/24)
Tsukamoto et al. [19]	2013	Japan	83	NA	>5% (48/35)	P (12/36) N (12/23)	NA	P (38/10) N (32/3)	P 10.4% (5/48) N 17.1% (6/35)
Lee et al. [28]	2014	Korea	67	Median 16.9 months	Score *⩾* 2 (36/31)	NA	NA	NA	P 11.1% (4/36) N 35.5% (11/31)
Jia [29]	2011	China	63	4–36 months	Score *⩾* 2 (47/16)	P (14/33) N (12/4)	P (31/16) N (16/10)	P (32/15) N (15/1)	P 0% (0/47) N 37.5% (6/16)
Liu [30]	2011	China	82	NA	Score *⩾* 2 (57/25)	P (16/41) N (16/9)	NA	P (37/20) N (24/1)	NA
Liu [31]	2004	China	45	NA	>20% (21/24)	P (3/18) N (12/12)	NA	P (10/11) N (20/4)	NA

## References

[1] Puleo F., Marechal R., Demetter P. (2015). New challenges in perioperative management of pancreatic cancer. *World Journal of Gastroenterology*.

[2] Hidalgo M. (2010). Pancreatic cancer. *The New England Journal of Medicine*.

[3] Stathis A., Moore M. J. (2010). Advanced pancreatic carcinoma: current treatment and future challenges. *Nature Reviews Clinical Oncology*.

[4] Ryan D. P., Hong T. S., Bardeesy N. (2014). Pancreatic adenocarcinoma. *The New England Journal of Medicine*.

[5] Moore B. W. (1965). A soluble protein characteristic of the nervous system. *Biochemical and Biophysical Research Communications*.

[6] Donato R. (2001). S100: a multigenic family of calcium-modulated proteins of the EF-hand type with intracellular and extracellular functional roles. *International Journal of Biochemistry and Cell Biology*.

[7] Salama I., Malone P. S., Mihaimeed F., Jones J. L. (2008). A review of the S100 proteins in cancer. *European Journal of Surgical Oncology*.

[8] Sherbet G. V., Lakshmi M. S. (1998). S100A4 (MTS1) calcium binding protein in cancer growth, invasion and metastasis. *Anticancer Research*.

[9] Helfman D. M., Kim E. J., Lukanidin E., Grigorian M. (2005). The metastasis associated protein S100A4: role in tumour progression and metastasis. *British Journal of Cancer*.

[10] Ismail N. I., Kaur G., Hashim H., Hassan M. S. (2008). S100A4 overexpression proves to be independent marker for breast cancer progression. *Cancer Cell International*.

[11] Rudland S. D. S., Martin L., Roshanlall C. (2006). Association of S100A4 and osteopontin with specific prognostic factors and survival of patients with minimally invasive breast cancer. *Clinical Cancer Research*.

[12] Liu Y., Tang W., Wang J. (2013). Clinicopathological and prognostic significance of S100A4 overexpression in colorectal cancer: a meta-analysis. *Diagnostic Pathology*.

[13] Lee S.-J., Choi S. Y., Kim W.-J. (2013). Combined aberrant expression of E-cadherin and S100A4, but not *β*-catenin is associated with disease-free survival and overall survival in colorectal cancer patients. *Diagnostic Pathology*.

[14] Kang Y.-G., Jung C.-K., Lee A., Kang W.-K., Oh S.-T., Kang C.-S. (2012). Prognostic significance of S100A4 mRNA and protein expression in colorectal cancer. *Journal of Surgical Oncology*.

[15] Wang Y.-Y., Ye Z.-Y., Zhao Z.-S., Tao H.-Q., Chu Y.-Q. (2010). High-level expression of S100A4 correlates with lymph node metastasis and poor prognosis in patients with gastric cancer. *Annals of Surgical Oncology*.

[16] Zhao Y., Zhang T., Wang Q. (2013). S100 calcium-binding protein A4 is a novel independent prognostic factor for the poor prognosis of gastric carcinomas. *Oncology Reports*.

[17] Xuan X., Li Q., Zhang Z., Du Y., Liu P. (2014). Increased expression levels of S100A4 associated with hypoxia-induced invasion and metastasis in esophageal squamous cell cancer. *Tumor Biology*.

[18] Chai J., Jamal M. M. (2012). S100A4 in esophageal cancer: is this the one to blame?. *World Journal of Gastroenterology*.

[19] Tsukamoto N., Egawa S., Akada M. (2013). The expression of S100A4 in human pancreatic cancer is associated with invasion. *Pancreas*.

[20] Yang H., Zhao K., Yu Q., Wang X., Song Y., Li R. (2012). Evaluation of plasma and tissue S100A4 protein and mRNA levels as potential markers of metastasis and prognosis in clear cell renal cell carcinoma. *Journal of International Medical Research*.

[21] Zhai X., Zhu H., Wang W., Zhang S., Zhang Y., Mao G. (2014). Abnormal expression of EMT-related proteins, S100A4, vimentin and E-cadherin, is correlated with clinicopathological features and prognosis in HCC. *Medical Oncology*.

[22] Ji Y.-F., Huang H., Jiang F., Ni R.-Z., Xiao M.-B. (2014). S100 family signaling network and related proteins in pancreatic cancer (review). *International Journal of Molecular Medicine*.

[23] Ling Z., Li R. (2014). Clinicopathological and prognostic value of S100A4 expression in gastric cancer: a meta-analysis. *International Journal of Biological Markers*.

[24] Wang Y., Zhou L.-B., Li X.-H. (2014). S100A4 expression and prognosis of gastric cancer: a meta-analysis. *Genetics and Molecular Research*.

[25] Bai H., Qian J.-L., Han B.-H. (2014). S100A4 is an independent prognostic factor for patients with lung cancer: a meta-analysis. *Genetic Testing and Molecular Biomarkers*.

[26] Oida Y., Yamazaki H., Tobita K. (2006). Increased S100A4 expression combined with decreased E-cadherin expression predicts a poor outcome of patients with pancreatic cancer. *Oncology Reports*.

[27] Ai K.-X., Lu L.-Y., Huang X.-Y., Chen W., Zhang H.-Z. (2008). Prognostic significance of S100A4 and vascular endothelial growth factor expression in pancreatic cancer. *World Journal of Gastroenterology*.

[28] Lee S. H., Kim H., Hwang J.-H. (2014). CD24 and S100A4 expression in resectable pancreatic cancers with earlier disease recurrence and poor survival. *Pancreas*.

[29] Jia F. X. *Expression and significance of S100A4, survivin and Cox-2 in pancreatic carcinoma tissues [Ph.D. thesis]*.

[30] Liu H. F. *The expression and significance of S100A4, MMP-2 and E-cad protein in pancreatic cancer [Ph.D. thesis]*.

[31] Liu A. A. *Expression of protein S100A4 and nm-23H1 in pancreatic ductal adenocarcinoma and its clinical significance [Ph.D. thesis]*.

[32] Ikenaga N., Ohuchida K., Mizumoto K. (2009). S100A4 mRNA is a diagnostic and prognostic marker in pancreatic carcinoma. *Journal of Gastrointestinal Surgery*.

[33] Li C. S., Ni C. R. (2006). Expression of S100A4 and E-cadherin in pancreatic carcinoma and their relationship study. *Chinese Journal of Clinical Hepatology*.

[34] Lu L., Ai K., Huang X. Y., Chen W., Zhang H. Z. (2008). Expression of S100A4 in pancreatic cancer tissue and its significance. *Journal of Oncology*.

[35] Lu L.-Y., Ai K.-X., Huang X.-Y., Chen W., Zhang H.-Z. (2008). Correlation of both S100A4 and VEGF expression with the prognosis of pancreatic cancer patients. *Tumor*.

[36] Jia F.-X., Liu J.-W., Zhang D., Li P., Li J.-Y., Lu K.-B. (2011). Correlation of both S100A4 and MMP-9 expressions with the prognosis of pancreatic cancer patients. *Chinese Journal of Cancer Prevention and Treatment*.

[37] Qin L. *Clinicopathological study and the expression of S100A4 in pancreatic cancer [Thesis]*.

[38] Jia F. X., Liu J., Zhang D., Li P., Li J. Y., Lu K. P. (2011). Correlation of both S100A4 and surviving expressions with the prognosis of pancreatic cancer patients. *Chinese Journal of Clinicians (Electronic Edition)*.

[39] Dahlmann M., Okhrimenko A., Marcinkowski P. (2014). RAGE mediates S100A4-induced cell motility via MAPK/ERK and hypoxia signaling and is a prognostic biomarker for human colorectal cancer metastasis. *Oncotarget*.

[40] Klingelhöfer J., Møller H. D., Sumer E. U. (2009). Epidermal growth factor receptor ligands as new extracellular targets for the metastasis-promoting S100A4 protein. *The FEBS Journal*.

[41] Donato R., Cannon B. R., Sorci G. (2013). Functions of S100 proteins. *Current Molecular Medicine*.

[42] Kimura K., Endo Y., Yonemura Y. (2000). Clinical significance of S100A4 and E-cadherin-related adhesion molecules in non-small cell lung cancer. *International Journal of Oncology*.

[43] Klingelhöfer J., Grum-Schwensen B., Beck M. K. (2012). Anti-S100A4 antibody suppresses metastasis formation by blocking stroma cell invasion. *Neoplasia*.

[44] Grum-Schwensen B., Klingelhöfer J., Beck M. (2015). S100A4-neutralizing antibody suppresses spontaneous tumor progression, pre-metastatic niche formation and alters T-cell polarization balance. *BMC Cancer*.

[45] Che P., Yang Y., Han X. (2015). S100A4 promotes pancreatic cancer progression through a dual signaling pathway mediated by Src and focal adhesion kinase. *Scientific Reports*.

[46] Sekine H., Chen N., Sato K. (2012). S100A4, frequently overexpressed in various human cancers, accelerates cell motility in pancreatic cancer cells. *Biochemical and Biophysical Research Communications*.

[47] Li N., Song M. M., Chen X. H., Liu L. H., Li F. S. (2012). S100A4 siRNA inhibits human pancreatic cancer cell invasion in vitro. *Biomedical and Environmental Sciences*.

[48] Tabata T., Tsukamoto N., Fooladi A. A. I. (2009). RNA interference targeting against S100A4 suppresses cell growth and motility and induces apoptosis in human pancreatic cancer cells. *Biochemical and Biophysical Research Communications*.

[49] Mahon P. C., Baril P., Bhakta V. (2007). S100A4 contributes to the suppression of BNIP3 expression, chemoresistance, and inhibition of apoptosis in pancreatic cancer. *Cancer Research*.

